# Co-Utilization of Sewage Sludge and Rice Husk in Ceramsite Preparation with Selective Adsorption Capacity to Pb

**DOI:** 10.3390/ma15124310

**Published:** 2022-06-17

**Authors:** Rui Wang, Meng Lu, Junxing Wang

**Affiliations:** 1 State Key Laboratory of Water Resources and Hydropower Engineering Science, Wuhan University, Wuhan 430072, China; whu_wangrui@foxmail.com; 2 Engineering Research Centre for Clean Production of Textile Dyeing and Printing, Ministry of Education, Wuhan Textile University, Wuhan 430073, China; lumeng27@163.com

**Keywords:** sewage sludge, rice husk, ceramsite, adsorption, Pb

## Abstract

Realizing the green recycling of sludge is an important link to effectively solve the problem of sludge disposal. In this paper, sewage sludge (SS) and rice husk (RH) were utilized as raw materials in preparing novel ceramsite (SRC) for the treatment of lead-containing wastewater, and its adsorption mechanism was explored. The results showed that the optimal preparation conditions were 40% RH + 60% SS mixture, a sintering temperature of 1190 °C, and a sintering time of 20 min. The basic properties of SRC met Chinese artificial ceramsite filter material standards for water treatment (CJ/T 299-2008). Under optimum adsorption conditions (pH = 6, 1 g/L SRC dosage, 20 mg/L Pb(NO)_3_ concentration, 18 h), the removal rate of Pb^2+^ reached 94.7%, and the equilibrium adsorption capacity was 18.94 mg/g. The adsorption process was more consistent with the pseudo-second-order kinetic model and the Langmuir isotherm model, indicating that the adsorption process was dominated by chemisorption. Thermodynamic parameters (ΔH^0^ > 0, ΔG^0^ < 0, ΔS^0^ > 0) indicated that the adsorption reaction was spontaneous and endothermic. The possible adsorption mechanisms are as follows: (1) SRC is rich in layered mesoporous structure, which provides sufficient reaction sites for Pb adsorption; (2) the sintered lawsonite and muscovite can strongly attract Pb and then form a new phase (Pb_10_[Si_2_O_7_]_3_(OH)_2_); (3) Pb^2+^ can bond with the Si–O- bond in aluminosilicates, and the introduction of Pb elevates the degree of polymerization of aluminosilicates in turn, indicating that the adsorption process is stable.

## 1. Introduction

With the development of industry, heavy metal pollution is intensifying, which poses a serious threat to environmental safety [1]. Pb pollution is one of the most common heavy metal pollution, which may cause neurological damage to humans and other creatures, damages the immune system, and even leads to death [2]. Among the many methods for disposing of Pb pollution, adsorption has been regarded as a promising method for the immobilization and recovery of Pb because of its high efficiency, easy operation, low cost of use, and so on [3].

On the other hand, the rate of urban sewage treatment has steadily improved as the economy has grown and urbanization has accelerated. As an unavoidable by-product of the sewage treatment process, the amount of sewage sludge (SS) has increased rapidly. For example, China has more than 5000 plants for sewage treatment and produces over 60 million tons of SS each year [4]. SS contains a lot of organic pollutants, heavy metals, and pathogens [5], hence the disposal of SS has thus become a critical environmental issue that must be addressed [6]. Incineration has emerged as the most potent SS treatment method in China because of the benefits of greatly lowering the volume and weight of SS, destroying bacteria, viruses, and organic impurities, and recovering thermal energy [7]. However, a large amount of ash containing a large amount of heavy metals is produced in the incineration process, which required further treatment and disposal [8,9]. Therefore, direct resource utilization may be a more effective means to SS disposal [10].

In recent years, some studies have attempted to use SS as raw materials in preparing ceramsite for building materials or filter materials [11,12]. Compared with the utilization of ceramsite in building materials, the higher added value of filter materials might be a more promising direction [13,14]. For example, Gong Cheng et al. [15] synthesized ceramsite from fly ash and SS to remove cadmium and copper in water. Wang et al. [16] produced ceramsite for phosphorus disposal in building wetlands using SS as the major raw material.

As reported by previous study [17], the Si/Al ratio has a considerable impact on the characteristics of ceramsite. Another study [18] also reported that the Si/Al ratio significantly affects the adsorption performance of the adsorbent. For instance, Alvarado-Perea et al. [19] reported that materials with higher Si/Al ratio exhibit higher specific surface area and better adsorption capacity. Therefore, additional materials (with high Si content) are necessary to adjust the composition of raw materials in order to obtain ceramsite with high adsorption performance. Silicon-containing auxiliary material is a good additive. Jing Nie et al. [20] added bentonite into SS to prepare ceramsite, and the obtained ceramsite were used to remove lead from wastewater. Changhui Wang et al. [21] used clay to improve the mechanical and adsorption properties of SS-based ceramsite for water treatment. However, most of the applied materials used in the above studies are inorganic components. The introduction of inorganic components might have an inhibitory effect on the formation of porous structures, which will weaken the adsorption capacity in turn. As a typical agricultural waste [22], rice husk (RH) has a high organic and silicon content [23]. This means RH might be used as additional material to provide Si and abundant organic matter during the preparation of SS-based ceramsite. As a hypothesis, RH-SS ceramsite may have good adsorption capacity and service characteristics at the same time. However, little research has been conducted on the use of RH as an additive in synthesizing SS-based ceramsite. The adsorption mechanism of this novel ceramsite product has not been studied.

Therefore, the purpose of this paper is to synthesize ceramsite (SRC) with excellent adsorption effects by SS and RH. The basic performance parameters of the selected SRC products were evaluated. Then, the corresponding adsorption mechanism was revealed by combining relevant detection means.

## 2. Materials and Methods

### 2.1. Materials

The SS was obtained in a sewage treatment plant in Wuhan, Hubei Province, China. RH was obtained in rural areas of Wuhan, Hubei Province, China. For subsequent usage, the SS and RH were dried to a consistent weight in a 105 °C oven and crushed using a 100-mesh standard sieve. The typical characteristics of the SS and RH are shown in Table 1. All the texts in this paper were repeated three times and the standard deviation was calculated by STDEV function. The contents of C and O in RH were substantially greater than those in SS. SS had high ash (71.51%) content and low volatility (27.61%), and its main inorganic components were SiO_2_, Al_2_O_3_, P_2_O_5_, and Fe_2_O_3_. Among these, SiO_2_ and Al_2_O_3_ were the main components of the framework of ceramsite, whereas Fe_2_O_3_, as the high temperature foaming component, contributed to the development of the porous structure [24]. Meanwhile, the organic content of RH was up to 85.83%. The inorganic composition of RH was mainly SiO_2_ (up to 83.81%). Therefore, the addition of RH could improve the Si/Al ratio and provide abundant organic matter. The proper addition of RH may help to form a well-developed framework [25]. However, excessive RH can lead to structural collapse. The appropriate RH content must thus be determined by further experiments.

According to Riley’s clay expansion theory [26], the main components of raw materials used to prepare ceramsite should be characterized by one of the following and the typical ceramsite phase will be obtained by high temperature combustion:

(1) (SiO_2_ + Al_2_O_3_)/(Fe_2_O_3_ + RO + R_2_O) = 3.5–10;
(2) SiO_2_: 48–70%; Al_2_O_3_: 8%–25%; and (Fe_2_O_3_ + CaO + MgO + K_2_O + Na_2_O): 4.5–31%.

Where RO stands for magnesium oxide and calcium oxide. R_2_O stands for sodium oxide and potassium oxide.

Considering the given raw material proportion requirements, the RH content may range from 0% to 70%.

### 2.2. Sample Preparation

Previous studies have shown that the sintering temperature [27] and sintering time [28] were the key factors affecting the properties of ceramsite. As a consequence, three independent variables (RH concentration, sintering temperature, and sintering duration) were chosen in this paper. Response surface methodology (RSM) based on the Box–Behnken design (BB) of three variables was adopted to optimize the preparation process of SRC. In order to estimate experimental errors and evaluate the applicability of the proposed model, 17 groups of experiments were performed, including 5 central points and 12 factorial points. The specific experimental design conditions are shown in Table S1. A, B, and C stand for the RH content, the sintering temperature, and the sintering time, respectively.

The specific experimental procedures were as follows:

(1) Pellet: SS and RH were mixed sufficiently according to their corresponding proportions (0%RH, 35%RH, and 70%RH) using a pelletizer to produce SRC raw pellets with diameters ranging from 5 to 10 mm. SRC raw pellets were obtained after natural air drying at room temperature for 3 days [29].
(2) Sintering: The SRC raw pellets were heated from the base temperature of 25 °C and then with a heating rate of 10 °C/min, the pellets were further heated to the preheating temperature (500 °C), and maintained for 20 min [30]. Finally, the SRC pellets were heated to the final temperatures (1050 °C, 1100 °C, and 1150 °C) at the same rate, and naturally cooled to room temperature after a period of sintering (10, 20, and 30 min) to obtain the final SRC products.
(3) Adsorption: The maximum Pb^2+^ removal rate was taken as the evaluation index to optimize the preparation process. The adsorption conditions were a 2 g/L dosage of SRC and a 20 mg/L concentration of Pb(NO)_3_. The adsorption equation was fitted according to the experimental results, and the analysis of variance (ANOVA) was conducted to investigate the suitability of the regression model. The optimum preparation process was selected according to the fitted equation, and the experimental adsorption rate was compared with the predicted value of the equation.

### 2.3. SRC Characteristics

The breaking and wear rate, silt carrying capacity, solubility in hydrochloric acid, and void fraction were tested according to the Chinese ceramsite filter material standards (CJ/T 299-2008). The BET of SRC was tested with automatic specific surface and porosity analyzer (Micrometrics, ASAP 2020 HD88, Norcross, GA, USA). The nitrogen adsorption and desorption curves were plotted, and the pore structure distribution and characteristics of SRC were analyzed. Meanwhile, the micromorphology of SRC was also evaluated using the scanning electron microscopy (SEM, Hitachi S-3500N, Tokyo, Japan) and X-ray computed tomography (X-ray CT, Xradia 510 Versa, Oberkochen, Germany).

### 2.4. Adsorption Experiments

Experiments on single-factor static adsorption were conducted to evaluate the effects of the SRC dosage, initial concentration of Pb(NO)_3_, pH, and adsorption time on the removal rate. The optimum adsorption conditions were then determined. Different concentrations (10, 20, 30, 40, 50, 60, and 70 mg/L) of the Pb(NO)_3_ solution were prepared to simulate actual wastewater, and the pH (3, 4, 5, 6, 7, 8, 9, 10, and 11) was regulated using 0.01 moles per liter Nitric acid solution or Sodium hydroxide solution. Adsorption was conducted in a horizontal vibration chamber at different time (1, 2, 3, 4, 6, 8, 10, 14, 18, and 24 h). The concentration of lead solutions was spotted using a flame atomic absorption spectrophotometer (AAS, Jena novAA800, Jena, Germany). The removal rate was calculated [31] as follows:

(1) $$R=\frac{C_{0}-C_{e}}{C_{0}}\times 100\%$$

where *R* stands for the removal rate, and *C*_0_ and *C_e_* stand for the initial and final Pb^2+^ concentration of the solution, respectively.

### 2.5. Adsorption Kinetics

Using pseudo-first-order and pseudo-second-order kinetic adsorption models, the adsorption kinetics of SRC were studied. Equations (2) and (3) show the equations [32] of the pseudo-first-order and pseudo-second-order models:

(2) $$\ln\left(q_{e}-q_{t}\right)=\ln q_{e}-k_{1}t$$

(3) $$\frac{t}{q_{t}}=\frac{1}{k_{2}q_{e}^{2}}+\frac{t}{q_{e}}$$

where *q_e_* (mg/g) stands for the adsorption capacity, *q_t_* (mg/g) stands for the adsorption capacity at time *t*, and *k*_1_ (1/min) and *k*_2_ (g/mg·min) stand for the adsorption rate constants, respectively.

### 2.6. Adsorption Isothermal

The equilibrium concentration relationship between the adsorbate and adsorbent was analyzed and calculated using the Langmuir and Freundlich isothermal models. The nonlinear fitting equations [33] of the two models are shown in Equations (4) and (5):

(4) $$q_{e}=\frac{q_{m}K_{L}C_{e}}{1+K_{L}C_{e}}$$

(5) $$q_{e}=K_{F}C_{e}^{1/n}$$

where *q_e_* stands for the adsorption capacity, *C_e_* stands for the equilibrium concentration, *q_m_* stands for the maximum adsorption capacity in theory, *K_L_* stands for the adsorption energy constant of Langmuir model, and *K_F_* stands for the adsorption capacity constant of Freundlich model.

### 2.7. Adsorption Thermodynamics

The influence of different temperatures on the adsorption process was analyzed in a thermodynamic study. The thermodynamic parameters, including the standard Gibbs free energy (ΔG^0^), the standard enthalpy change (ΔH^0^), and the standard entropic change (ΔS^0^) were calculated and the calculation equations [34] are expressed in Equations (6)–(8):

(6) $$K_{d}=\frac{q_{e}}{C_{e}}$$

(7) $${\Delta G}^{0}={\Delta H}^{0}-T{\Delta S}^{0}$$

(8) $$\ln K_{d}=\frac{{\Delta S}^{0}}{R}-\frac{{\Delta H}^{0}}{RT}$$

where *q_e_* stands for the adsorption capacity, *C_e_* stands for the equilibrium concentration, and *R* (8.3145 J/mol·K) is the ideal gas constant.

### 2.8. Mineral Composition

X-ray diffraction (XRD, Bruker advance D8, Berlin, Germany) was used to characterize the mineral compositions of the synthesized SRC before or after adsorption. Before loading the SRC into the test platform, it was ground and passed through a 200-mesh standard screen.

### 2.9. Mineral Composition

X-ray photoelectron spectroscopy (XPS, ESCALAB 250XI, Waltham, MA, USA) was used to determine the binding energies of Si, Al, O, and Pb before and after adsorption. By analyzing the transformation of chemical bond after the adsorption of Pb, the mode of action of Pb adsorption by SRC was conjectured.

## 3. Results and Discussion

### 3.1. Response Surface Methodology

**Figure 1:**
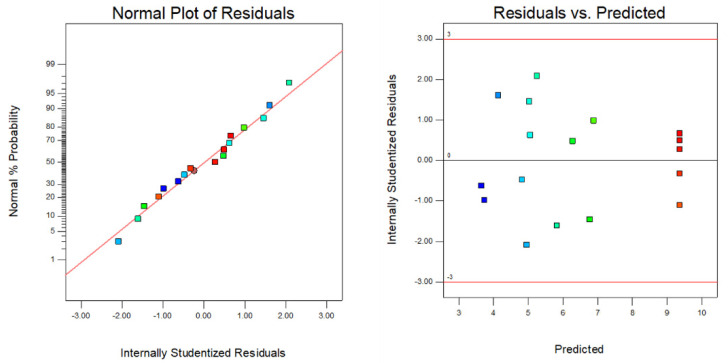
Normal distribution validation and residuals versus predictions.

Response results of the adsorption capacity under different conditions are shown in Table S2. Table 2 summarizes the ANOVA results for the response surface models. The model was statistically significant since “Prob > F” had a value less than 0.001 [35]. The lack of fit had a *p* value that was more than 0.05, which indicated that the lack of fit was not significant in comparison to the levels of pure error. The determination coefficient *R*^2^ was 0.9909, implying that 99.09% of the variance could be explained by the model and that the model can reliably describe the relationship between the adsorption efficiency and the SRC preparation process [36]. Figure 1 shows the residuals distribution of the model. Normal distribution of the residuals implied that the model was significant. Since residuals were uniformly distributed on both sides of the directrix, the suggested model was near to reality [37].

The adsorption efficiency of SRC prepared under different conditions can be expressed as the following optimized relationships:

(9) $$\begin{array}{l} \mathrm{Adsorption}\;\mathrm{efficiency}\\ =9.36+0.86A-0.71B+0.013C-0.10\mathrm{AB}+0.46\mathrm{AC}-0.60\mathrm{BC}-1.83A^{2}-2.32B^{2}\\ -2.09C^{2} \end{array}$$

**Figure 2:**
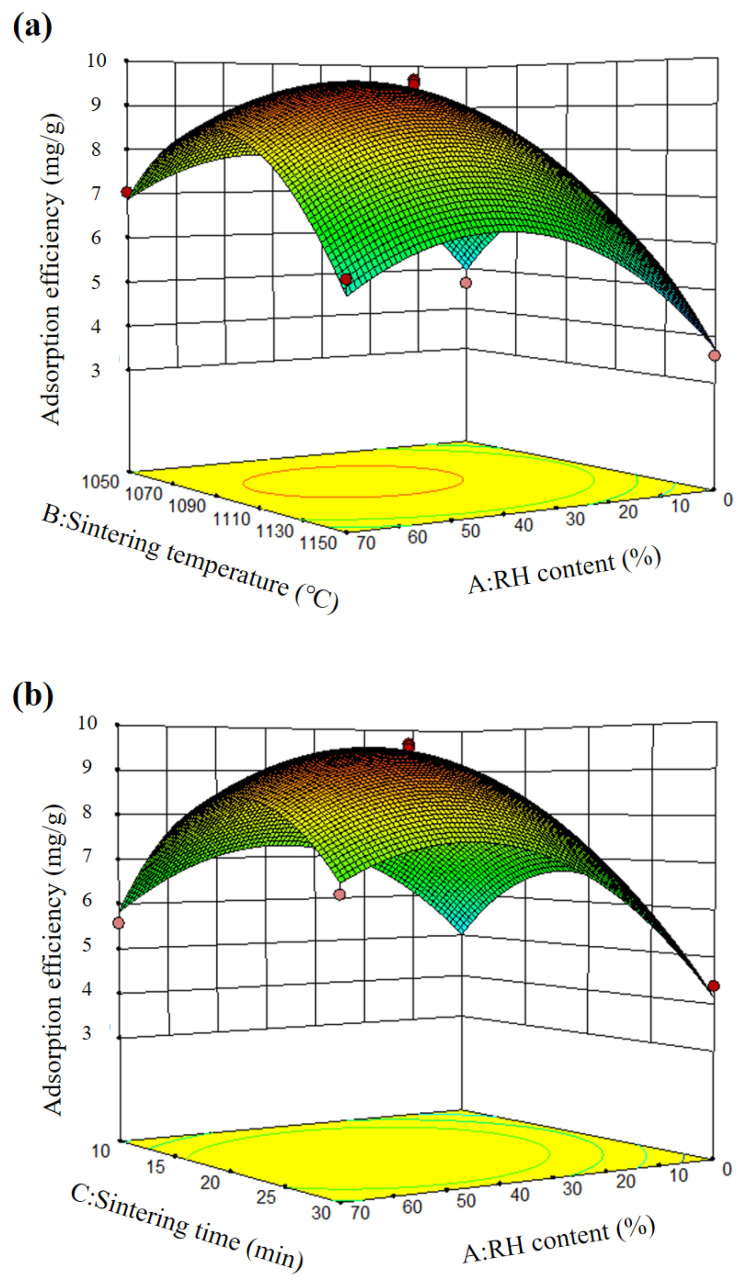

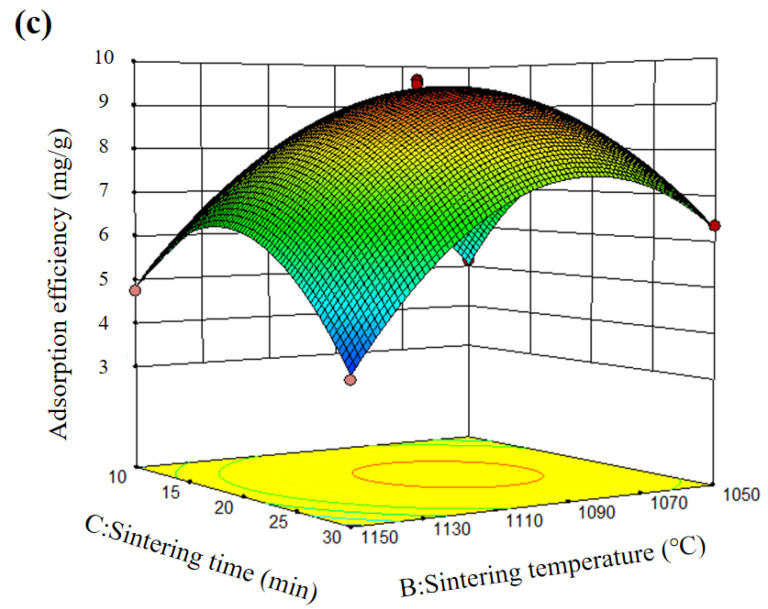
Response surface diagram of (**a**) A × B, (**b**) A × C, and (**c**) B × C.

The synergistic impact of these independent factors was visually represented by using the three-dimensional response surfaces predicted by the regression models. Synergistics between each pair of variables were studied. Figure 2 shows the three-dimensional response surfaces.

Figure 2a depicts the response surface between RH concentration and sintering temperature. The figure demonstrates that the adsorption efficiency improved during the first increase in temperature and subsequently declined as the temperature continued to rise when the RH concentration remained constant. This is probably because: when the temperature was low, the positive mineral phase and pore structure have not been formed; when the temperature was high, excess molten phases are formed which will plug the mesoporous spaces, and then the porosity of the SRC products was reduced [38]. It is noteworthy that by deriving the combustion temperature from the adsorption efficiency, it was found that the RH content affected the derivative (DAS) of adsorption efficiency to sintering temperature. At a low dosage (20%), the DAS decreased rapidly with the temperature increase below peak value. In contrast, at a high dosage (70%), the decrease rate of the DAS slowed down obviously when temperature increased below peak value. This phenomenon is because RH can not only be utilized in the capacity of a pore-forming agent, but also improve the melting point of the system [39]. It is conducive to the formation of porous structures and finally promote the adsorption process. Therefore, the decrease in the adsorption effect caused by the increase in temperature was partially offset.

Figure 2b shows the response surface between the RH content and the sintering time. When the RH content was low (20%), the adsorption efficiency decreased from 5.256 mg/g to 4.385 mg/g as sintering time increased from 10 to 30 min. On the contrary, when the RH content was high (70%), the adsorption efficiency increased from 5.578 mg/g to 6.548 mg/g as the sintering duration increased. The reason for this difference may be the two main factors controlling the pore structure in this system: (1) the promotion effect, where the pore-increasing effect of RH; (2) the inhibition effect, where the pores collapse with the extension of the sintering time. Under low RH content, inhibition controlled the process, whereas at high content, the promotion effect was dominant.

Figure 2c shows the response surface between the sintering temperature and the sintering time. As temperature and time rose, the adsorption efficiency increased and then declined. When the sintering temperature was relatively low (1050 °C), the adsorption efficiency increased from 5.153 mg/g to 6.352 mg/g with the increase in the reaction time from 10 to 30 min. When at relatively high temperatures (1150 °C), the adsorption efficiency decreased from 4.749 mg/g to 3.556 mg/g as sintering duration increased from 10 to 30 min. This is because the reaction was slow at low temperatures, and extending the sintering time helped the adequate reaction. However, the reaction was violent at high temperatures, and the structural collapse induced by over sintering became more obvious as the time was extended.

From the above analysis, it can be obtained that the appropriate RH content is 40%, the appropriate sintering temperature is 1190 °C, and the appropriate sintering time is 20 min. Under optimum condition, the maximum adsorption capacity obtained by the experiment was 9.618 mg/g, which was close to the predicted value of 9.527 mg/g, which verified the reliability of the prediction model.

### 3.2. SRC Characteristics

The basic characteristics of the SRC products are shown in Table 3. The breaking and wear rate of the SRC was 3.9%, the silt carrying capacity was 0%, the solubility in hydrochloric acid was 0.31%, the void fraction was 56.4%, and the total BET surface area was 46,824 cm^2^/g. All the above parameters met the requirements of the Chinese Industrial standards (CJ/T 299-2008).

**Figure 3:**
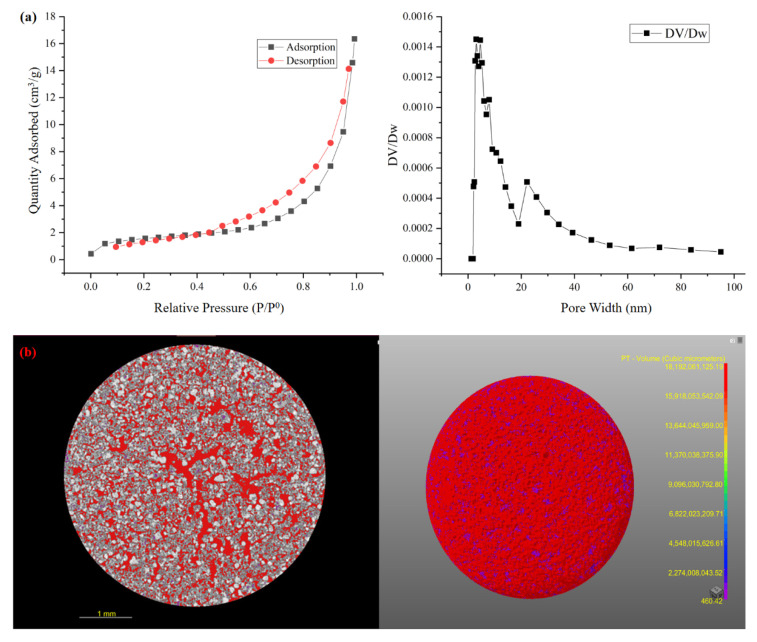
Pore structure characterization of RSC: (**a**) The N_2_ adsorption/desorption isotherms of SRC and the pore size distribution, and (**b**) CT.

Figure 3 shows the pore structure characteristics of the SRC products. As illustrated in Figure 3a, the N_2_ adsorption/desorption isotherm of SRC according to the IUPAC classification was a typical type-IV curve with an H3-type hysteresis loop. The type-IV isotherm indicated that SRC processes the mesoporous structure, while the H3-type hysteresis loop indicated that the mesopores existed as interconnected pore structure, which was beneficial for the adsorption performance [40]. Besides, the pore size distribution curve also shows that the pores in SRC products were mainly mesopores (2 < pore width < 50 nm), and only a small part were macropores (pore width > 50 nm). Studies have shown that the developed mesoporous structure can provide sufficient reaction sites, which is conducive to adsorption [41]. CT (Figure 3b) shows that the SRC has a rich pore structure and the pore size is uniform. Moreover, corresponding to the bet result, these holes are interconnected.

### 3.3. Static Adsorption of Pb

#### 3.3.1. Adsorption Single-Factor Experiment

**Figure 4:**
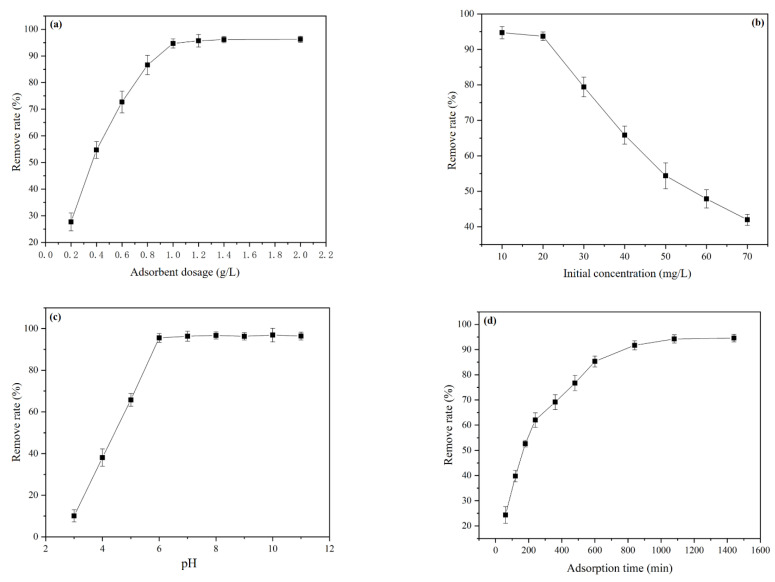
Single factor adsorption results: (**a**) adsorbent dosage and remove rate, (**b**) initial concentration and remove rate, (**c**) pH and remove rate, and (**d**) time and remove rate.

The effect of the amount of adsorbent on the removal rate of Pb is shown in Figure 4a. With the increase in the dosage of SRC, the remove rate of Pb^2+^ increased significantly. When the dosage of the SRC reached 1 g/L, almost all Pb^2+^ (94.7%) in the 20 mg/L lead solution were removed, continuous increase of adsorbent dosage had no significant effect on the increase of removal rate. Consequently, 1 g/L is the optimal dose of the adsorbent from the standpoint of efficient resource consumption.

Figure 4b shows the effect of the initial adsorbate concentration on the removal rate. When the concentration of the adsorbate ranged from 10 to 20 mg/L, the removal rate of 1 g/L of the adsorbent can reach more than 94%. With the continuous increase in Pb(NO)_3_ concentration, the Pb^2+^ removal rate decreased gradually. When the adsorbate concentration reached 30 mg/L, the removal rate was only 79.4%, which was 14.3% lower than that at 20 mg/L. Thus, selecting the initial adsorbate concentration of 20 mg/L is appropriate.

Figure 4c demonstrates the effect of pH on SRC adsorbent capacity. With pH rising, the adsorption capability of SRC improved considerably. When the pH reached 6 or above, the remove rate reached its maximum value, and the Pb^2+^ removal rate reached more than 94%. This may be because when the pH was low, there were numerous free hydrogen ions in the system, and the diffusion of lead ions to the surface of the SRC filter material was hindered by electrostatic repulsion [42], which in turn prevented effective adsorption. Under alkaline conditions (pH > 6), the removal of Pb^2+^ was mainly due to the formation of Pb(OH)_2_ precipitates [31]. In alkaline conditions, the vast majority of Pb is removed as precipitates, which is not meaningful for the evaluation of adsorption characteristics of materials. Therefore, pH = 6 is a reasonable adsorption condition.

The effect of time on the removal rate of Pb is shown Figure 4d. As time increased, the removal rate increased gradually, the adsorption process reached equilibrium when the time reached about 18 h, and the removal rate reached above 94%. Therefore, the subsequent experiments considered that the adsorption equilibrium can be reached at 18 h.

In conclusion, the optimum Pb^2+^ adsorption conditions on the SRC are as follows: pH = 6, 1 g/L SRC dosage, 20 mg/L Pb(NO)_3_ concentration, and 18 h adsorption time. Under these conditions, the removal rate was 94.7%, and the equilibrium adsorption capacity was 18.94 mg/g.

#### 3.3.2. Adsorptive Kinetics

**Figure 5:**
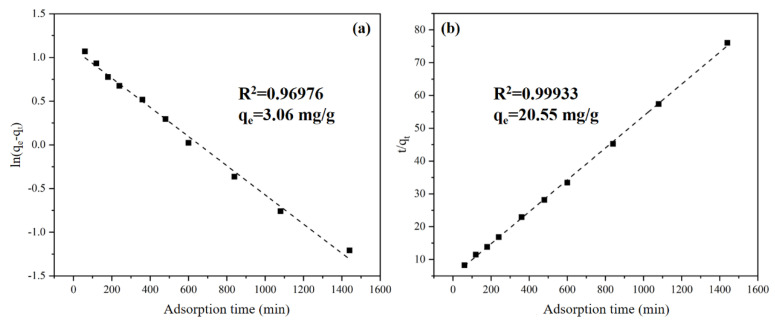
Fitting line of pseudo-first order (**a**) and pseudo-second order (**b**).

Figure 5 displays the linear fitting outcomes of the pseudo-first-order and pseudo-second-order adsorption kinetic models of the SRC to Pb^2+^. The R^2^ value of first-order model was 0.97, and the generated equilibrium adsorption capacities of 3.06 mg/g; whereas the R^2^ value of pseudo-second-order kinetic model exceeded 0.999 with the fitted equilibrium adsorption capacities of 20.57 mg/g. Comparing the equilibrium adsorption capacities produced by model fitting, it was evident that the value generated by the second-order model was closer to the experimental value (18.9 mg/g), indicating that the Pb^2+^ adsorption process on the SRC is more consistent with the second-order fitting model. The second-order kinetic model assumes that the adsorption process is a process with a limited adsorption rate, with the adsorption rate mainly influenced by chemical mode, including the ion exchange and covalent bonding [34]. That meant the adsorption mechanism of SRC to Pb was mostly chemisorption.

#### 3.3.3. Adsorption Isothermal

**Figure 6:**
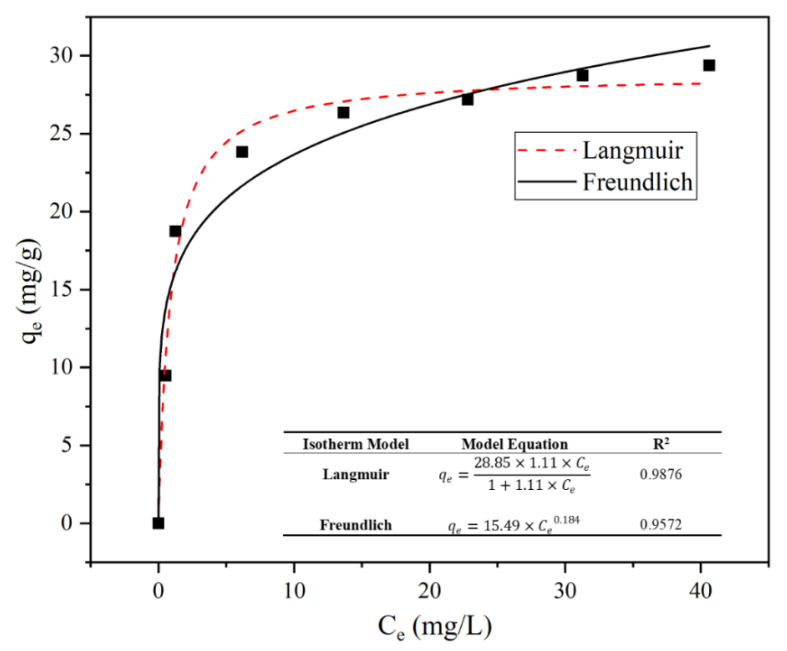
Isothermal fitting of Langmuir and Freundlich equations.

Nonlinear fitting was performed to examine the adsorption process using the Langmuir and Freundlich isothermal adsorption models. As shown in Figure 6, the Langmuir model more suitably described the isothermic adsorption process due to its higher *R*^2^ (0.9876). This means that the adsorption process was monolayer and homogenous, where all sorption sites were equivalent and energetically identical [43]. Based on Langmuir model, the maximum theoretical adsorption capacity of SRC to Pb was calculated to be 28.85 mg/g, which was extremely similar to the experimental value (29.38 mg/g). These results proved that the model was applicable. It is worth mentioning that the Freundlich model also had a high confidence level (*R*^2^ > 0.95). The parameter 1/*n* calculated by the Freundlich model represented the degree of difficulty in the reaction–adsorption process. The small value of 1/*n* (0.184 < 0.5) indicated that the adsorption reaction occurred easily [44].

#### 3.3.4. Adsorption Thermodynamics

**Figure 7:**
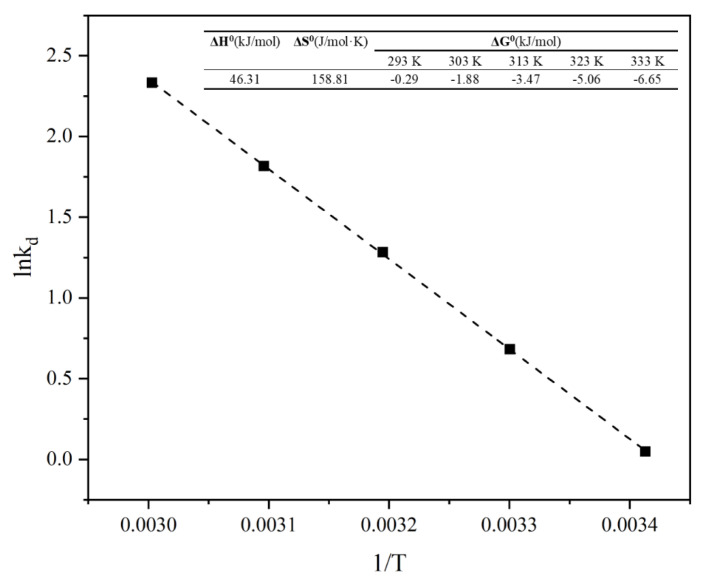
Adsorption thermodynamics.

Thermodynamic parameters at different temperatures from 293 K to 333 K were calculated. ΔH^0^ and ΔS^0^ were calculated using the slope and the *y*-axis intercept of the plot of the ln*K*_d_ versus 1/*T* curve, whereas ΔG^0^ was calculated by ΔH^0^ and ΔS^0^ (Figure 7). 

Positive ΔH^0^ value indicated that the adsorption reaction was endothermic. High temperature was favorable to the adsorption process and, in other words, the equilibrium adsorption capacity increase with increasing temperature [45]. The ΔH^0^ value (20.9 < ΔH^0^ < 418.4 kJ/mol) of SRC indicated that the adsorption process was dominated by chemical adsorption [46], which is consistent with the kinetics analysis (Section 3.3.2). Wu et al. [47] reported the characteristics of positive ΔS^0^ and small ΔH^0^ meant the existence of electrostatic attraction in the adsorption process. It could be assumed that physisorption caused by electrostatic attraction may also exist in this study. Other studies have shown that physical adsorption may also occur simultaneously during chemisorption-dominated adsorption processes [48]. The negative value of ΔG^0^ indicated that the adsorption of Pb^2+^ by SRC was spontaneous [49]. ΔG^0^ decreased from −0.29 to −6.65 kJ/mol as the temperature increased from 293 K to 333 K, indicating that the rise in temperature facilitated the adsorption response [50].

### 3.4. Possible Adsorptive Mechanisms

#### 3.4.1. XRD Analysis

**Figure 8:**
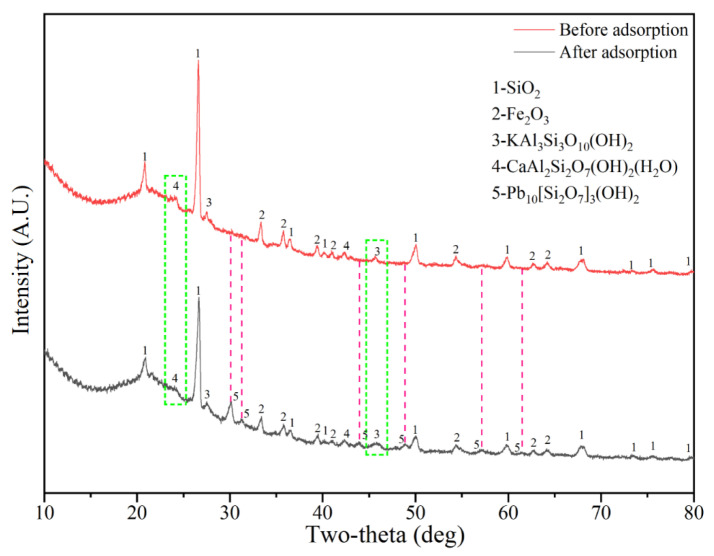
XRD analysis before and after adsorption.

Mineral composition analyses before and after the adsorption of SRC are shown in Figure 8. Typical quartz and hematite were detected in SRC, whereas lawsonite (CaAl_2_Si_2_O_7_(OH)_2_(H2O)) and muscovite (KAl_3_Si_3_O_10_(OH)) were also found. Lawsonite and muscovite were formed during sintering where silicon and potassium were mainly provided by RH, whereas aluminum and calcium were mainly provided by SS. López et al. [51] found that the lawsonite structure could easily bond with lead and form a Pb–lawsonite structure. Di et al. [52] reported that the muscovite surface is permanently negatively charged because of the isostructural substitution of Al^3+^ for Si^4+^, which may have a strong adsorption effect on heavy metal cations through electrostatic interactions. A new phase, Pb_10_[Si_2_O_7_]_3_(OH)_2_, was detected in the SRC after adsorption. After adsorption, the peak of lawsonite at a 2*θ* of 24.03° disappeared, showing that lawsonite participated in the chemical reaction during adsorption to generate the new mineral phase. Meanwhile, the peak of muscovite at a 2*θ* of 45.7° changed from a single peak to a flat inclusion peak after adsorption, most likely due to Pb^2+^ entering the muscovite lattice and replacing a part of the skeleton atoms, which made the original mineral tend to be amorphous [41]. These findings indicated that Pb was immobilized in the SRC matrix through ion exchange with aluminosilicate minerals, which was consistent with the conjecture obtained from adsorption kinetics studies.

#### 3.4.2. XPS Analysis

**Figure 9:**
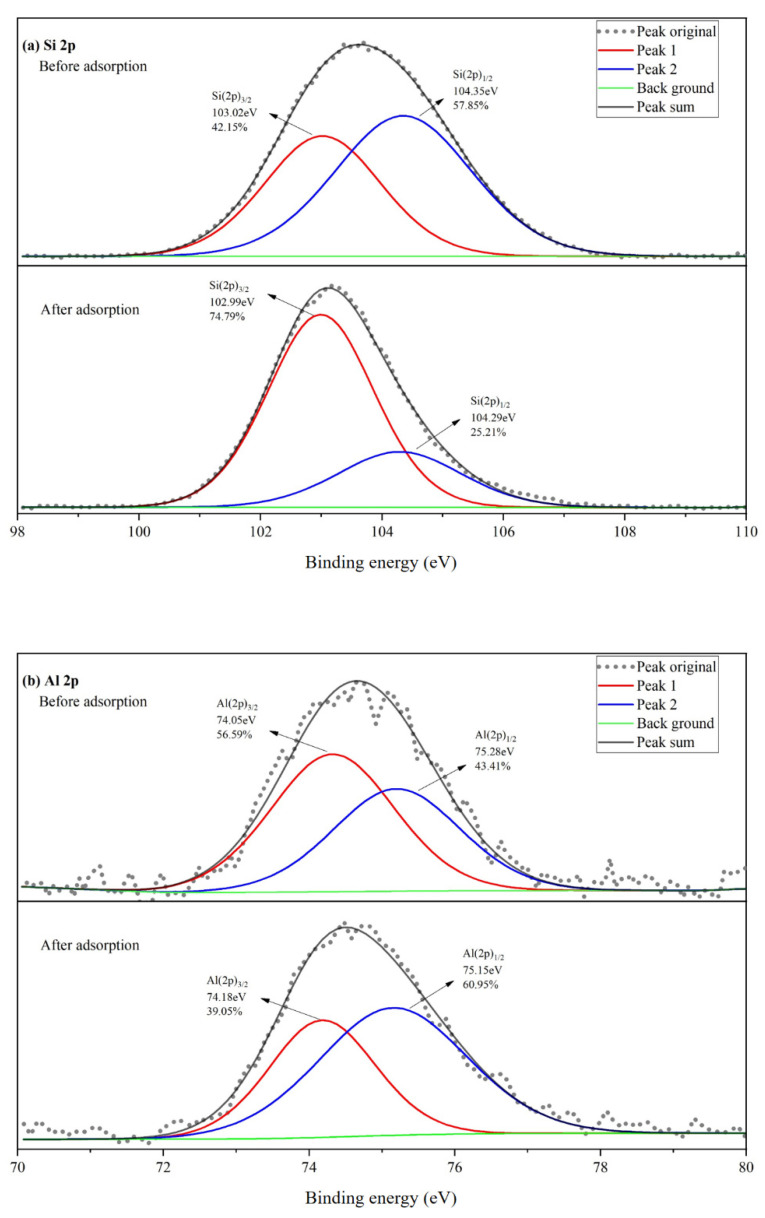

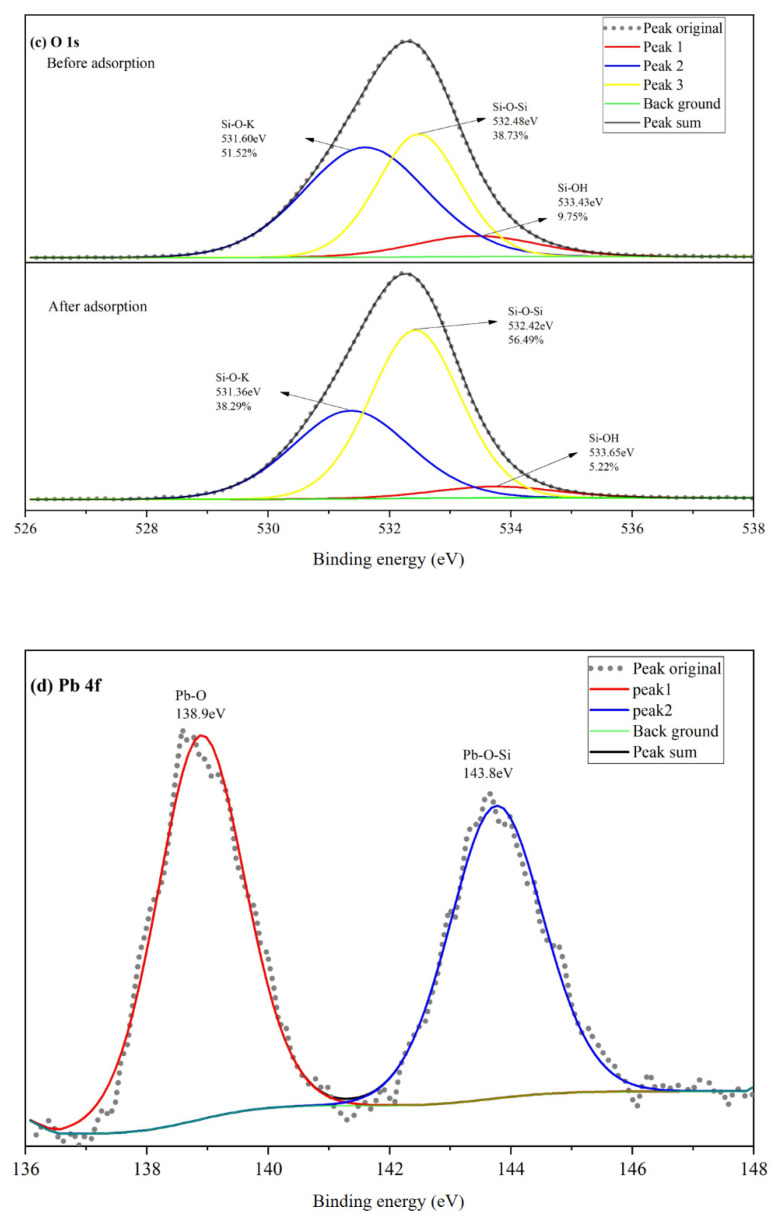
XPS spectra of SRC before and after adsorption: (**a**) Si 2p, (**b**) Al 2p, (**c**) O 1s, and (**d**) Pb 4f.

XPS was employed to analyze the relative chemical bonds intensity of Si 2p, Al 2p, O 1s, and Pb 4f orbitals in SRC before and after adsorption, and the results are shown in Figure 9. According to C–C (284.8 eV), all peak positions were adjusted. The binding energies of Si 2p and Al 2p of SRC before and after adsorption are shown in Figure 9a,b, respectively. The signals of Si 2p and Al 2p can be divided into two peaks. For Si 2p signal, the peaks of 103.02 eV and 102.99 eV were attributed to zeolite serials while the peaks of 104.35 eV and 104.29 eV were related to quartz component [53]. For Al 2p signal, the peaks of 74.05 eV and 74.08 eV represented octahedral aluminum and the peaks of 75.28 eV and 75.15 eV stands for tetrahedron aluminum [54]. The ratio of Si(2p)_3/2_ increased from 42.15% to 74.79% after adsorption, indicating chemical reactions occurred between SRC and Pb, and then crystalline structure transformed to zeolite-like mineral phase. However, lead-containing zeolites were not found according to the XRD results, probably because the adsorption time was short and not enough to produce the lead-containing zeolite mineral phase, while existing in a zeolite-like state. Chen et al. [55] reported that lead-containing zeolites could be formed after about 3 days of reaction, and this conclusion could verify our deduction. After adsorption, the ratio of Al(2p)_3/2_ decreased from 56.59% to 39.05% while Al(2p)_1/2_ increased from 43.41% to 60.95%, indicating there were more tetrahedral aluminum generating in the adsorbed SRC. Namely, the SRC matrix became more polymerized after adsorption. Combined XPS results of Si 2p and Al 2p in the overall, the invasion of Pb^2+^ destroyed the original chemical bonds in the system and formed zeolite-like bonds in the SRC to form more polymerized structure.

The O 1s bond before and after adsorption can be analyzed as shown in Figure 9c. There are three peaks in the binding energy of O 1s: the peaks of 531.60 and 531.36 eV were related to Si–O–K, the peaks of 532.48 and 532.42 eV represented Si–O–Si, and the peaks of 533.43 and 533.65 eV were attributed to Si–OH [56]. The ratio of Si–O–Si after adsorption increased from 38.73% to 56.49%, which indicated that the intrusion of Pb^2+^ caused more aggregation of the SRC’s components, consistent with the conclusions of the Si 2p and Al 2p analysis. The decreased ratio of Si–O–K and Si–OH indicated that these two bonds from aluminosilicate minerals might have reacted with Pb^2+^ to form new bonds. For example, in combination with the XRD analysis, Pb likely reacted with muscovite and replaced K in Si–O–K of the muscovite to form Si–O–Pb bonds.

The characteristic peak of Pb 4f was detected in the adsorbed SRC, as shown in Figure 9d. The peak at 143.8 eV corresponded to Si–O–Pb, and the peak at 138.9 eV was attributed to Pb–O [57,58]. The existence of the Si–O–Pb bond verified the conjecture that ion exchange takes place between Pb and Si–O–K. The existence of the Pb–O bond means there were free Pb–O not solidified into the mineral but attached to the surface of the SRC, which proved that physisorption and chemisorption worked simultaneously. In this system, Pb was pulled and reacted with aluminosilicate in the SRC to form Si–O–Pb and solidified in the geopolymer structure at first. Then, the excess Pb existed in the free state (Pb–O). This may be due to the limited Pb^2+^ immobilization ability of the SRC lattice. Other studies also reached similar conclusions [55].

## 4. Conclusions

The optimum preparation parameters of the SRC determined using RSM are as follows: the RH content is 40%, the appropriate sintering temperature is 1190 °C, and the appropriate sintering time is 20 min. The removal rate of Pb^2+^ reached 96.18% at an SRC dosage of 2 g/L and Pb(NO)_3_ concentration of 20 mg/L. The basic performance parameters of the SRC products were as follows: the breaking and wear rate was 3.9%, the silt carrying capacity was 0%, the solubility in hydrochloric acid was 0.31%, the void fraction was 56.4%, and the total BET surface area was 46,824 cm^2^/g. These met the Chinese artificial ceramsite filter material standards for water treatment (CJ/T 299-2008). Under the optimum adsorption conditions (pH = 6, 1 g/L SRC dosage, 20 mg/L Pb(NO)_3_ concentration, 18 h), the removal rate of Pb^2+^ reached 94.7%, and the equilibrium adsorption capacity of SRC was 18.94 mg/g. The adsorption process was more consistent with the pseudo-second-order kinetic model, indicating that the adsorption process was more likely dominated by chemisorption. The *R*^2^ value of the Langmuir isotherm model was higher than Freundlich model, indicating the adsorption process was monolayer adsorption and that each adsorption site was equivalent. Thermodynamic parameters (ΔH^0^ > 0, ΔG^0^ < 0) indicated that the adsorption reaction was spontaneous and endothermic. The positive value of ΔS^0^ indicated that physisorption also existed in the adsorption process. The possible adsorption mechanisms are as follows: (1) SRC is rich in layered mesoporous structure, which provides sufficient reaction sites for Pb adsorption; (2) the sintered lawsonite and muscovite can strongly attract Pb and then form a new phase of Pb_10_[Si_2_O_7_]_3_(OH)_2_; (3) Pb^2+^ can bond with the Si–O- bond in aluminosilicates, and the introduction of Pb elevates the degree of polymerization of aluminosilicates in turn, indicating that the adsorption process is stable.

## Figures and Tables

**Table 1:** Typical characteristics of raw materials.

	Component	RH (%)	SS (%)
**Proximate analysis**	Ash	14.17 ± 0.10	71.51 ± 0.74
	Volatile matter	71.41 ± 0.29	27.61 ± 0.31
	Fixed carbon	14.42 ± 0.21	0.88 ± 0.05
**Ultimate analysis**	C	41.68 ± 0.74	12.04 ± 0.11
	H	5.76 ± 0.08	1.31 ± 0.01
	O	37.68 ± 1.89	13.38 ± 1.55
	N	0.71 ± 0.03	1.76 ± 0.15
**Ash analysis**	Al_2_O_3_	0.64 ± 0.03	16.49 ± 0.43
	SiO_2_	83.81 ± 1.79	50.55 ± 0.89
	Fe_2_O_3_	2.63 ± 0.06	7.78 ± 0.17
	Na_2_O	0.18 ± 0.01	0.53 ± 0.04
	K_2_O	5.62 ± 0.14	3.00 ± 0.01
	CaO	1.63 ± 0.12	4.60 ± 0.17
	P_2_O_5_	0.48 ± 0.02	11.78 ± 0.91
	MgO	0.71 ± 0.04	2.65 ± 0.05

**Table 2:** Analysis of variance of response surface regression model.

Source	Sum of Squares	Df	Mean Square	F Value	*p*-Value Prob > F
Model	73.90	9	8.21	84.62	<0.0001
A	5.92	1	5.92	61.03	0.0001
B	4.08	1	4.08	42.03	0.0003
C	0.0014	1	0.0014	0.014	0.9085
AB	0.042	1	0.042	0.44	0.5296
AC	0.85	1	0.85	8.73	0.0213
BC	1.43	1	1.43	14.47	0.0064
A^2^	14.13	1	14.13	145.62	<0.0001
B^2^	22.69	1	22.69	233.79	<0.0001
C^2^	18.36	1	18.36	189.16	<0.0001
Lack of Fit	0.52	3	0.17	4.27	0.0972
Std. Dev.	0.31	*R* ^2^	0.9909	Adj R^2^	0.9792
Mean	6.42	Adeq Precision	23.892		

**Table 3:** Physical characteristics of ceramsite.

Parameters	Ceramsite	Criterion
**Breaking and wear rate (%)**	3.9 ± 0.5	≤6
**Silt carrying capacity (%)**	N.d	≤1
**Solubility in hydrochloric acid (%)**	0.31 ± 0.06	≤2
**Void fraction (%)**	56.4 ± 1.4	≥40
**Water absorption (%)**	61 ± 4	\
**Specific surface area (×10^4^, cm^2^/g)**	4.68 ± 0.18	≥0.5
**Total pore volume (cm^3^/g)**	0.024 ± 0.004	\
**Average pore diameter (nm)**	20.7 ± 1.6	\

## Supplementary Materials

The following supporting information can be downloaded at: https://www.mdpi.com/article/10.3390/ma15124310/s1, Table S1: Factors and levels of Box–Behnken design; Table S2: Experimental design and the corresponding results.
