# Supplementary material for: Co-Utilization of Sewage Sludge and Rice Husk in Ceramsite Preparation with Selective Adsorption Capacity to Pb
Source: Materials (Basel). 2022 Jun 17;15(12):4310. doi: 10.3390/ma15124310 (PMC9230551; doi:10.3390/ma15124310)
Supplement: Supplementary file 1 [file materials-15-04310-s001.zip › materials-1757738-supplementary.pdf]

Table S1. Factors and levels of Box-Behnken design

Level	RH content (%)	Sintering temp (°C)	Sintering time (min)
-1	0	1050	10
0	35	1100	20
1	70	1150	30

Table S2. Experimental design and the corresponding results

.Group	Experimental design			Response
	A	B	C	Adsorption efficiency (mg/g)
1	1	1	0	5.577
2	0	0	0	9.271
3	-1	1	0	3.584
4	0	-1	-1	5.153
5	0	0	0	9.546
6	0	1	-1	4.749
7	-1	-1	0	4.634
8	1	0	-1	5.578
9	1	0	1	6.548
10	0	-1	1	6.352
11	1	-1	0	7.039
12	0	0	0	9.499
13	-1	0	-1	5.256
14	-1	0	1	4.385
15	0	0	0	9.054
16	0	0	0	9.438
17	0	1	1	3.556
